# Effects of citrulline on endurance performance in young healthy adults: a systematic review and meta-analysis

**DOI:** 10.1080/15502783.2023.2209056

**Published:** 2023-05-08

**Authors:** Callum S. Harnden, JOSEPH Agu, TOM Gascoyne

**Affiliations:** University of Nottingham, Department of Sports and Exercise Medicine, Faculty of Medicine and Health Sciences, Nottingham, UK

**Keywords:** Citrulline, watermelon, endurance, exercise, aerobic

## Abstract

**Background:**

Citrulline is a popular dietary supplement, primarily thought to exert ergogenic effects on exercise performance through the enhancement of nitric oxide (NO) synthesis and ammonia buffering. However, recent findings surrounding citrulline’s effect on endurance performance have been inconsistent. A systematic review and meta-analysis of the relevant literature have yet to be undertaken.

**Aim:**

To determine if acute ingestion of citrulline has an ergogenic effect on endurance performance in young healthy adults.

**Methods:**

A systematic search of three databases was undertaken to find peer-reviewed randomized controlled trials (RCTs) published in English investigating the effects of citrulline supplementation on endurance performance in young healthy adults. Two independent investigators completed a three-phased screening procedure against pre-determined eligibility criteria. Included studies evaluated loading or bolus dosage regimes of citrulline in participants aged 18 or over that were at least recreationally active. Outcome measures focused on time-to-completion (TTC) or time-to-exhaustion (TTE) in continuous submaximal intensity exercise. Cochrane’s Risk of Bias 2 (RoB 2) tool was used to assess the risk of bias in individual studies. Meta-analysis was conducted using a fixed-effects model to pool the weighted estimate of standardized mean differences (SMD) across studies. A chi-squared test assessed heterogeneity between studies. This review was conducted and reported in accordance with the Preferred Reporting Items for Systematic Reviews and Meta-Analyses (PRISMA) guidelines.

**Results:**

Nine studies (*n* = 158 participants) met the eligibility criteria; five reported TTE outcomes (I^2^ = 0%, χ^2^ = 0.37, df = 4, *P* = 0.99) and four reported TTC outcomes (I^2^ = 0%, χ^2^ = 0.46, df = 3, *P* = 0.93), both with a low between-study heterogeneity. The results of the meta-analyses showed no significant difference in the endurance performance measures, TTE (pooled SMD = 0.03 [−0.27, 0.33]) and TTC (pooled SMD = −0.07 [−0.50, 0.15]), after acute ingestion of citrulline supplementation or a control in young healthy adults.

**Discussion:**

The current evidence suggests no significant benefit of citrulline supplementation for endurance performance. However, the small evidence base requires further research to fully evaluate this topic. Recommendations include a focus on female populations; higher continuous doses of citrulline over seven days; and TTC outcome measures over longer distances to simulate competition.

## 1. Introduction

L-citrulline is an increasingly popular dietary supplement used for performance enhancement in multiple areas of exercise. It is a non-essential, non-protein amino acid compound most abundantly found in watermelon [1]. The two principal metabolic pathways involving citrulline are the urea cycle and the nitric oxide (NO) cycle [2]. Within the NO cycle, citrulline is a by-product of arginine-dependent NO synthesis by nitric oxide synthase (NOS) [3]. Citrulline is indirectly produced from arginine via ornithine in the sequestration of ammonia in the urea cycle [3]. In both processes, citrulline is converted back to arginine via arginosuccinate in a dual enzymatic process [1,3].

Increased levels of plasma citrulline enhance plasma arginine and ornithine concentrations. Plasma concentrations of citrulline seem to be the primary determinant of citrulline conversion to arginine [4]. The enhancement of plasma arginine concentration is purported to have many ergogenic effects. A growing interest in citrulline supplementation stems from findings of its ability to increase plasma arginine levels to a greater extent than supplementation with arginine itself [5,6]. This is because citrulline is more readily absorbed in the gut and bypasses first-pass metabolism unlike arginine [5,7]. Most ingested citrulline is metabolized to arginine by arginosuccinate synthase and arginosuccinate lyase in the proximal tubules of the kidney [8,9].

The primary theory by which citrulline supplementation is believed to exhibit an ergogenic effect is via enhancement of NO synthesis. This effect causes endothelium-dependent, NO-mediated vasodilation, which is hypothesized to increase blood flow to contracting muscles, improving oxygen delivery, and the capacity for aerobic respiration [10]. Enhancement of oxygen delivery to contracting muscles potentially reduces any limitative effects this factor has on maximal oxygen uptake (VO_2_ max) [11,12]. Another hypothesis states that citrulline’s augmentation of NO bioavailability potentiates the enhancement of muscle oxygenation and speeds up VO_2_ kinetics [13]. This is founded on reports that the rate of increase in VO_2_ is restricted by the ability to deliver oxygen to contracting muscles upon exercise commencement [14]. VO_2_ kinetics are slower than the immediate increase in metabolism observed in active muscles at the beginning of exercise [15]. Therefore, anaerobic processes are also required to meet energy demand, resulting in the early production of fatigue-developing metabolites [15]. Ashley et al. found that seven days of l-citrulline supplementation improved VO_2_ kinetics during walking in men, but not women [15]. Similarly, Bailey et al. found the overall VO_2_ kinetics were faster during severe-intensity exercise following a seven-day supplementation period of 6 grams of citrulline compared to a placebo [16].

An alternative mechanism proposed surrounds citrulline’s role in buffering ammonia through the urea cycle [17]. Greater ammonia accumulation is associated with muscular fatigue, which may greatly diminish endurance performance [18,19]. Raised blood levels of ammonia promote anaerobic glycolysis, increasing blood lactate levels [20]. Elevated levels of ammonia and lactate in the muscle cause accumulation of hydrogen ions, reducing the pH and impeding muscular contraction [20]. Therefore, by reducing levels of ammonia, citrulline supplementation theoretically should augment the aerobic utilization of pyruvate. In turn, lessening anaerobic respiration and lactate production, preventing impedance of muscular contraction [17,21]. Inhibition of lactate accumulation may prolong exercise at a set VO_2_, delaying breach of lactate and anaerobic thresholds. Inconsistency exists in the literature regarding the elicitation of an effect of citrulline on blood lactate levels after exercise [17,22–26]. L-citrulline is commonly combined with malic acid to form citrulline malate (CM). Malate is also thought to exhibit restrictive effects on lactic acid accumulation, though there is limited evidence solely investigating its effects [1].

Regardless of the purported physiological mechanisms surrounding citrulline supplementation, the exhibition of an ergogenic effect on exercise performance within the literature is inconsistent. The majority of research underlying citrulline supplementation focuses on power and strength outcomes in resistance exercises. Trexler et al. undertook a systematic review and meta-analysis summarizing the effects of citrulline supplementation on high-intensity power and strength outcomes [27]. The results showed a significant benefit of ingesting citrulline compared to placebo treatments, with a small pooled SMD of 0.2 (95% confidence intervals = 0.01 to 0.39). Varvik et al. also performed a meta-analysis finding a small ergogenic effect of CM compared with placebo on repetition performance outcomes during strength training [28].

Aerobic endurance is the ability of the cardiorespiratory system to efficiently supply metabolic substrate to working muscles in sustained submaximal intensity physical activity [29]. Endurance performance is determined by three principal factors: VO_2_ max, the lactate threshold (LT), and the power output efficiency [30]. VO_2_ max is primarily restricted by the cardiovascular system’s aptitude to deliver oxygen rather than the oxygen extraction capabilities of the contracting muscles [11,12]. Central limiting factors include pulmonary diffusing capacity, maximal cardiac output, and the blood’s oxygen-carrying capacity [11]. Peripheral influences surround skeletal muscle characteristics, such as peripheral diffusion gradients, mitochondrial enzyme levels, and capillary density [11].

Hickner et al. conducted the first study investigating the effects of citrulline supplementation on TTE in submaximal intensity exercise in healthy individuals [31]. Comparing the effects of 3 and 9 grams of l-citrulline to placebo on a graded treadmill test in physically active males and females. A negative effect was reported, finding that treadmill TTE was significantly lower following citrulline ingestion. Contrastingly, Bailey et al. found that a 7-day supplementation period of 6 grams of l-citrulline per day, significantly improved performance in a severe intensity cycling TTE test compared to a placebo in recreationally active males. Other studies measured endurance performance velocity in time trials, using time-to-completion (TTC) as an outcome measure over a set distance. Suzuki et al. reported that a 7-day supplementation period of l-citrulline significantly reduced the time taken to complete a cycle ergometer exercise trial compared to a placebo. Another study using a similar supplementation protocol reported no significant difference in the time taken to complete a 40-kilometer cycle [17].

A comprehensive systematic review and meta-analysis to summarize the literature surrounding acute citrulline supplementation and endurance performance has yet to be undertaken. The ambiguity of findings presented in the literature merits such a process. Gonzalez et al. recently summarized some of the key findings as part of a literature review looking at the effects of citrulline supplementation on exercise performance ADDIN EN.CITE <EndNote><Cite><Author>Gonzalez</Author><Year>2020</Year><IDText>Effects of Citrulline Supplementation on Exercise</IDText><DisplayText> [32]</DisplayText><record><titles><title>Effects of Citrulline Supplementation on ExercisePerformance in Humans: A Review of the Current Literature.</title><secondary-title>The Journal of Strength and Conditioning Research</secondary-title></titles><pages>1480–1495</pages><number>5</number><contributors><authors><author>Gonzalez, AM.</author><author>Trexler, ET.</author></authors></contributors><added-date format=“utc”>1595499026</added-date><ref-type name=“Journal Article”>17</ref-type><dates><year>2020</year></dates><rec-number>1349</rec-number><last-updated-date format=“utc”>1595499169</last-updated-date><volume>34</volume></record></Cite></EndNote> [32]. Fernández-Hernández et al. published a systematic review in Spanish investigating the effects of l-citrulline in endurance sports but did not perform a meta-analysis [33]. Both reviews included studies that did not directly utilize outcome measures associated with aerobic endurance performance. One study observed outcomes using maximal exertion rather than submaximal exertion [23] and two others in the later review used biomarkers rather than performance measures [6,7]. Another study also included in the later review used a combination of supplementation rather than citrulline alone as the intervention [34]. Fernández-Hernández et al. did not include multiple RCTs that evaluated the effects of citrulline supplementation of endurance performance [24,35]. Additionally, since the publication of these reviews, additional research has emerged, warranting further review using a more thorough systematic approach [36,37].

The purpose of performing this systematic review and meta-analysis is to determine if acute ingestion of citrulline has an ergogenic effect on endurance performance in young healthy adults. RCTs assessing the effects of acute citrulline supplementation on performance in continuous submaximal intensity exercise in young healthy adults will be utilized in this review. It is hypothesized that there is no difference in endurance performance after acute ingestion of citrulline compared with a control.

## 2. Methods

### 2.1. Protocol and registration

A copy of the study protocol is accessible on request from CH or JA. This protocol was not registered prior to the undertaking of this systematic review it.

### 2.2. Eligibility criteria

A strict set of inclusion and exclusion criteria using a PICO design were established to ensure all included studies were relevant to the review’s question. Studies were included if they: (a) were peer-reviewed, published in English and reported in full-text format; (b) reported data from young healthy adults aged 18 years or over who at least recreationally participate in exercise; (c) reported quantitative data from RCTs sufficient for computation of effect sizes regarding the impact of citrulline supplementation on endurance performance; (d) administered any dosage of citrulline, as a loading regime or bolus at least 30 minutes prior to exercise to ensure sufficient time for absorption [5]; (e) delivered citrulline supplementation as l-citrulline, CM or l-citrulline contained within a watermelon juice drink; (f) used outcomes focusing on endurance performance in continuous submaximal intensity exercise, determined by measures surrounding performance velocity or time-to-exhaustion (TTE).

Studies were excluded if they: (a) were review articles, theses, dissertations, or unpublished; (b) delivered citrulline doses less than 30 minutes before exercise testing, preventing sufficient time for absorption; (c) used an intervention that involved mixing citrulline with other potentially ergogenic supplements; (d) used a form of citrulline supplementation that was not l-citrulline, CM, or citrulline containing juice mixes.

Varying dosages of citrulline have been utilized in previous studies, but currently, no minimum effective dosage for endurance performance has been identified [24]. Therefore, a minimum dosage has not been defined in the eligibility criteria. Citrulline supplementation has been shown to cause peak plasma citrulline concentration at approximately one hour and peak plasma arginine concentration at approximately two hours [5].

### 2.3. Information sources

To identify eligible published literature for this systematic review, the following bibliographic databases were searched through the respective interfaces in May 2020: MEDLINE (1966 – Present, OVID), EMBASE (1980 – Present, OVID), SPORTDiscus (1930 – Present, EBSCO). Retrospective citation chaining was completed using the reference lists from already obtained articles. The electronic table of contents of important journals and conference proceedings were used to manually search for studies. Two relevant researchers in the field were contacted to ascertain their awareness of relevant unpublished or recently submitted data. Grey literature was not searched for due to difficulties in locating it through systematic and transparent searching, and the increased risk of bias that the lack of peer review associates with it. These limitations of gray literature were deemed to outweigh the benefits, such as a reduction in publication bias, diversity of evidence sources, and identification of ongoing or unpublished studies.

### 2.4. Search

The search strategy used in this review follows PRISMA guidelines. The free-text and MeSH terms used in this search were developed through scoping searches, review of the relevant literature, and discussion with experts in the field. The following terms were used and appropriately adapted for the different interfaces and databases: “citrulline,” “l-citrulline,” “citrulline malate,” “citmal,” “watermelon” OR “l-arginine” in amalgamation with “endurance,” “aerobic,” “continuous exercise,” “submaximal intensity exercise,” “cycl*,” “run*,” “sport performance,” “exercise tolerance” OR “physical endurance.” Asterisks were used to widen the scope of the search to include, terms, such as “cycle,” “cycling,” “cyclists” as well as “run,” “running” and ”runners.” Unavailability of experts with specific language and database searching skills was one search constraint. However, the search strategies used in the systematic review process were peer-reviewed by TG and JA. Endnote was the bibliographic software used to store and manage search results. Searches were repeated in April 2022 to ascertain newly published studies.

### 2.5. Study selection

An electronic screening and selection tool was developed using the eligibility criteria to enhance the identification of relevant papers. Prior to use it was piloted and adapted to prevent regular disagreements later on in the screening process. In adherence to the PRISMA guidelines, after the removal of duplicates, a three-stage screening process was undertaken. Involving title and abstract screening, followed by full-text article reviews against the predetermined eligibility criteria. This was carried out by two independent investigators (CH and TG) and disagreement at any stage in the screening process was debated and resolved by the project supervisor (JA). Studies suitable for meta-analysis were discussed by the principal investigator (CH) and the project supervisor (JA). This meta-analysis only allowed for comparison between citrulline supplementation and control, other comparators in multiple treatment arm studies were not included. Studies reported in a non-English language were excluded due to the risk of introducing language bias as an accurate translation process was lacking.

### 2.6. Data collection process

Data extraction tables were initially piloted using a sample of the included studies to assess for the practicality of data extraction and completeness of captured data; they were refined accordingly. Two different types of data were collected, descriptive and analytical. Initial data extraction by the principal investigator (CH) was reviewed by an independent investigator (TG), any disputes were resolved by the project supervisor (JA). Electronic data extraction allowed data to be copied and pasted into the tables to reduce the risk of data-entry error. Extracted data was highlighted in the source papers to assist with rapid identification of its origin, allowing swift dispute resolution. No measures were undertaken to check or obtain missing characteristic data from study investigators, it was felt this would be a timely process that would not change the outcome.

### 2.7. Data items

The descriptive data collected includes study characteristics such as the first author; year published; source journal; study design; location of study; supplement dosage and form; dosage regime; comparators; performance test; outcome measure for endurance performance; the number of participants included in the analysis; and study sponsorship. Participant characteristics extracted include sample size (including dropouts); mean age; sex; and training level. The analytical data collected includes the group means plus the standard deviation (SD) or standard error (SE). No additional data variables were considered after the review process commenced to reduce the risk of reporting bias. Any studies not clearly defining the number of patients included in their analysis were assumed to have included all participants.

### 2.8. Risk of bias in individual studies

The included studies are all randomized controlled trials (RCTs) with a cross-over design; therefore, their methodological rigor were appropriately assessed using the RoB 2 tool proposed by Cochrane [38]. The risk-of-bias was assessed at a study and outcome level. Studies were judged to have a high risk, low risk, or some concerns of bias based on the five key domains displayed in Table 2. The tool included algorithms that assigned responses to signaling questions to propose a risk-of-bias judgment, these quality assessments were not cross-checked. The small number of included studies means that studies with some concerns or a high risk-of-bias were included in the quantitative synthesis. A post hoc sensitivity analysis was conducted, excluding the studies with a high risk-of-bias, to ascertain the robustness of the originally conducted meta-analyses.

**Table 1.** ~TC~

Study (first author, year)	Source	Study design	Location	Sample size (Dropouts)	Age, years (mean ± SD)	Sex	Training level	Supplement dosage and form	Dosage regime (loading -days/bolus: timing before exercise)	Comparators	Type of endurance exercise: performance protocol	Outcome of interest	Number of participants included in analyses	Study sponsorship (conflict of interest)	Study findings
Bailey et al. 2015	Journal of Applied Physiology	Randomized, double-blind, placebo-controlled, crossover	Exeter, United Kingdom	10	19 ± 1	M	Recreationally active	6g L-Citrulline	Loading − 7 days: 90 minutes before exercise	6g L-Arginine, Placebo	Cycling: severe intensity on day 7	Time-to-exhaustion	NS	ND	↑ TTE
Bailey et al. 2016	Nitric Oxide	Randomized, double-blind, placebo-controlled, crossover	Exeter, United Kingdom	8	22 ± 2	M	Recreationally active	300ml WJ (approximately 3.4g of L-Citrulline)	Loading − 16 days: 75 minutes	Placebo	Cycling: Moderate and severe intensity on day 14	Time-to-exhaustion	NS	ND	↔ TTE
Cutrufello et al. 2014	Journal of Sports Sciences	Randomized, double-blind, placebo-controlled, crossover	Louisville, USA	22: 11 male/11 female	20.6 ± 1.2 (male); 21.0 ± 1.3 (female)	Mix	Recreationally trained	6g L-Citrulline	Bolus: 10 participants 60 minutes and 12 participants 120 minutes	710ml WJ (approximately 1g of L-Citrulline), Placebo	Running: graded treadmill test	Time-to-exhaustion	22	ND	↔ TTE
Gills et al. 2020	European Journal of Sport Science	Randomized, double-blind, placebo-controlled, crossover	NS	28 [4]	20.9 ± 2.8	M	Recreationally trained (endurance-based exercise twice a week for >1 year)	8g CM	Bolus: 60 minutes	Placebo	Cycling: at 90% of previously recorded VO_2peak_ above 40 RPM pedalling cadence	Time-to-exhaustion	28	ND	↔ TTE
Hickner et al. 2006	Medicine & Science in Sports & Exercise	Randomized, double-blind, placebo-controlled, crossover	Greenville, USA,	17	NA (range 18–40 years old)	Mix	Physically active	3g or 9g L-Citrulline	Bolus: last meal before exercise	Placebo	Running: graded treadmill test	Time-to-exhaustion	17	ND	↓ TTE
Martínez- Sánchez et al. 2017	Food & Nutrition Research	Randomized, double-blind, placebo-controlled, crossover	Cartagena, Spain	21	35.3 ± 11.4	M	Amateur runners	WJ + L-Citrulline (approximately 6.91g L-Citrulline)	Bolus: 60 minutes	Placebo	Running: half-marathon time trial	Time to completion	NS	Ministerio de Economía y Competitividad; Fashion Group Association (ND)	↔ TTC
Shanely et al. 2016	Nutrients	Randomized controlled, crossover	North Carolina, USA	20	48.5 ± 2.3	M	Competitive cyclists	980mL WJ (approximately 1.47g L-Citrulline)	Loading − 14 days: ingested during time trial	No treatment plus 6% carbohydrate beverage during trial	Cycling: 75km time trial	Time to completion	NS	National Watermelon Promotion Board (One author is a science advisor to this Board)	↔ TTC
Stanelle et al. 2020	The Journal of Strength and Conditioning	Randomized, double-blind, placebo-controlled, crossover	Texas, USA	10	24 ± 3	M	Trained triathletes or cyclists	6g L-Citrulline	Loading − 7 days: 120 minutes	Placebo	Cycling: 40km time trial	Time to completion	9	The Sydney & J.L. Huffines Institute for Sports Medicine & Human Performance (ND)	↔ TTC
Suzuki et al. 2016	Journal of the International Society of Sports Nutrition	Double-blind, placebo-controlled, crossover	Osaka, Japan	22 [3]	29 ± 8.4	M	Trained	2.4g L-Citrulline	Loading − 8 days: 60 minutes	Placebo	Cycling: 4km time trial	Time to completion	22	KYOWA HAKKO BIO CO (Two authors are employees)	↓ TTC

SD standard deviation, NS not stated, USA United States of America, CM citrulline malate, M male, ND not declared.

↔ no significant difference (*p*>0.05) compared to placebo, ↑ significantly greater (*p*<0.05) than placebo, ↓ significantly less (*p*<0.05) than placebo.

**Table 2.** Results of risk of bias assessment using the ROB 2 tool.

Bias domain	Criteria within domain	Bailey et al. 2015	Bailey et al. 2016	Cutrufello et al. 2014	Gills et al. 2020	Hickner et al. 2006	Martínez- Sánchez et al. 2017	Shanely et al. 2016	Stanelle et al. 2020	Suzuki et al. 2017
Bias arising from the randomization process	Allocation sequence randomised?	PY	PY	PY	PY	PY	PY	PN	Y	Y
	Allocation sequence concealed?	PY	PY	PY	Y	PY	PY	NI	Y	Y
	Baseline imbalances suggesting problematic randomization?	NI	NI	NI	NI	N	NI	NI	N	NI
	Roughly equal proportion of participants allocated to each group?	NI	NI	NI	NI	Y	NI	NI	Y	NI
	Period effects included in analysis?	NI	NI	NI	NI	NA	NI	NI	NA	NI
	Overall risk of bias	Some concerns	Someconcerns	Some concerns	Someconcerns	Low risk	Some concerns	High risk	Low risk	Someconcerns
Bias due to deviations from intended interventions	Participants aware of assigned intervention during each trial period?	N	N	N	N	N	N	PY	N	N
	Trial personnel aware of assigned intervention during each trial period?	N	N	N	N	N	N	PY	N	N
	Deviations from intended interventions beyond usual practice?	NA	NA	NA	NA	NA	NA	PN	NA	NA
	Were deviations unbalanced between the two interventions?	NA	NA	NA	NA	NA	NA	PN	NA	NA
	Sufficient time for carry-over effects to disappear?	Y	Y	Y	Y	Y	Y	Y	Y	Y
	Overall risk of bias	Low risk	Low risk	Low risk	Low risk	Low risk	Low risk	Low risk	Low risk	Low risk
Bias due to missing outcome data	Outcome data available for all participants randomised?	PY	PY	Y	N	Y	PY	Y	Y	Y
	Are missing outcome data similar across interventions?	NA	NA	NA	Y	NA	NA	NA	NA	NA
	Results robust to presence of missing outcome data?	NA	NA	NA	Y	NA	NA	NA	NA	NA
	Overall risk of bias	Low risk	Low risk	Low risk	Low risk	Low risk	Low risk	Low risk	Low risk	Low risk
Bias in measurement of the outcome	Were outcome assessors aware of intervention allocation?	PN	PN	PN	PN	PN	PN	NI	N	PN
	Was outcome assessment likely to be influenced by knowledge of intervention received?	NA	NA	NA	NA	NA	NA	N	NA	NA
	Overall risk of bias	Low risk	Low risk	Low risk	Low risk	Low risk	Low risk	Low risk	Low risk	Low risk
Bias in selection of the reported result	Reported outcome data likely selected from multiple outcome measurements in the outcome domain?	N	N	N	N	N	N	N	N	N
	Reported outcome data likely selected from multiple analyses of the data?	N	N	N	N	N	N	N	N	N
	Reported outcome data likely selected from the outcome of a statistical test for carry-over?	N	N	N	N	N	N	N	N	N
	Overall risk of bias	Low risk	Low risk	Low risk	Low risk	Low risk	Low risk	Low risk	Low risk	Low risk
Overall risk of bias judgment		Some concerns	Some concerns	Some concerns	Some concerns	Low risk	Some concerns	High risk	Low risk	Some concerns

Y yes, PY probably yes, N no, PN probably no, NI no information, NA not applicable.

### 2.9. Summary measures

Studies presented continuous data separately for the intervention and control groups, providing a mean and SD or a mean and SE. All SE Data was converted to SD using the equation $SD=SE\times \sqrt{N}$ proposed by The Cochrane Collaboration [39]. The SD is the correct way to measure the data variability around the mean of a sample; SE measures the precision for an estimated population mean [40]. The SMD was calculated to standardize the results of the studies to a uniform scale. However, the SMD does not account for differences in the scale’s direction. Therefore, the two identified outcome measures were unable to be summarized together as TTE favors a higher number and TCC a lower number. Moreover, the comparability of TTE and time trial tests is debated within the literature [41–43].

The interpretation of this summary statistic should generally follow the rule that a small effect is represented by 0.2, a moderate effect by 0.5, and a large effect by 0.8 [44]. Subjectively, studies were deemed to be clinically and methodologically homogeneous, allowing data to be combined. The population, interventions, comparators, and outcomes were considered. Results described similar effects with overlapping of corresponding confidence intervals, further justifying the appropriateness of meta-analysis.

### 2.10. Synthesis of results

A meta-analysis was conducted using a fixed-effects model in Review Manager (RevMan) [Computer program]. Version 5.3. Copenhagen: The Nordic Cochrane Centre, The Cochrane Collaboration, 2014. This utilized the inverse variance method to pool the weighted estimate of SMD across studies. A chi-squared test for heterogeneity was performed; due to the small number of studies included and the small sample sizes of each, a p-value of 0.1 was used to determine significance [45]. The I^2^ statistic was used to determine the degree of any heterogeneity identified. Low risk of heterogeneity was determined by a value <25%, a moderate risk of heterogeneity by a value ranging from 25% to 75%, and a high risk by a value >75% [46]. Forest plots generated by the RevMan software were used to present the results of individual studies plus the overall combined estimate of the effect. These analyses were discussed with a specialized statistician when designing the review to ensure appropriateness.

### 2.11. Risk of bias across studies

It was decided not to generate a funnel plot and carry out tests for funnel plot asymmetry to assess for publication bias. Too few studies included in the meta-analyses would have rendered the power of the tests too low to distinguish chance from real asymmetry. Trial registries were inspected in an attempt to identify missing studies. Evidence of selective reporting was assessed through comparison with available study protocols and thorough examination of published articles to identify missing outcomes.

### 2.12. Additional analyses

Insufficient data prevented comparison between subgroups; therefore, subgroup analyses and meta-regression were not performed. One post hoc sensitivity analysis was conducted, excluding studies with a high risk of bias to validate the robustness of the original meta-analyses. All analyses were conducted by the principial investigator (CH).

## 3. Results

### 3.1. Study selection

**Figure 1.**
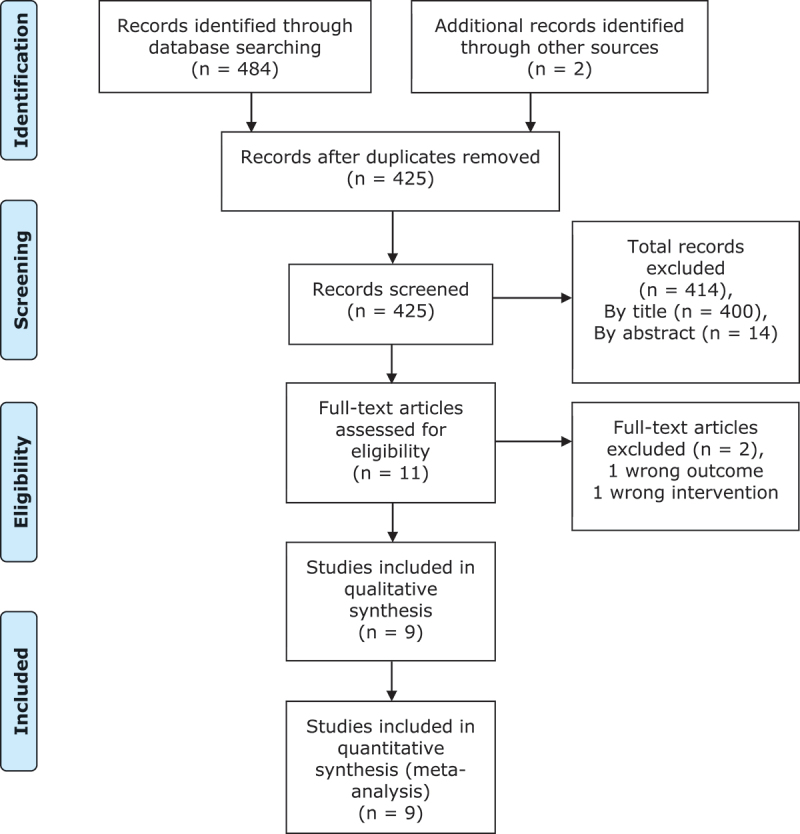
PRISMA flow diagram.

Database searching identified 484 studies; two additional studies were identified through citation chaining. After removing duplicates, 425 studies remained to be screened for inclusion. Firstly, titles and abstracts were screened to assess their relevance to the review question, 414 were deemed irrelevant, leaving 11 studies eligible for full-text review. Full texts were obtained for all 11 studies, two of these were excluded. One did not examine the appropriate intervention and one did not measure the correct outcome. No additional studies were identified following contact with experts in the field or manual searching. Nine studies were included in the systematic review, all of which were included in the quantitative synthesis. The PRISMA flow diagram for this systematic review is displayed in Figure 1. There were no disputes between the independent investigators at any point during the screening process, therefore no arbitration was required.

### 3.2. Study characteristics

The nine studies included were published between 2006 and 2020, their characteristics are summarized in Table 1. A randomized, double-blind, placebo-controlled, crossover design was used for all studies but one [35]. Shanely et al. used a randomized, crossover design but did not blind participants or researchers, using a nutrient matched 6% carbohydrate beverage as a control rather than a placebo [35]. Four studies were conducted in the United States of America [24,31,35,36], two in the United Kingdom, one in Spain [17], one in Japan [47], and one in an undefined location [37].

A total number of 158 participants were involved in this review; sample sizes of the included studies ranged from 8 to 28. All participants were healthy young adults, 18 years or older; the mean age of sample sizes varied with the majority falling between 18 and 30 years old [16,24,37,47,48]. Two studies had slightly older samples with mean ages of 35.3 ± 11.4 [17] and 48.5 ± 2.3 [35], another did not calculate a mean age but provided a range of 18–40 years old [31]. A variety of training levels were observed across the studies, from recreationally active to trained and competitive athletes. All samples were exclusively male asides from two studies that contained a mix of males and females [24,31], both combined results rather than presenting data individually by sex.

The most common form of supplementation was free-form l-citrulline, one study used CM, and three used watermelon juice. All watermelon juice supplements were tested to calculate their concentration of l-citrulline. The most common l-citrulline dose was 6 grams, although doses ranged from 1.47 to 9 grams. Studies either delivered supplementation using a loading phase plus a final bolus before testing [16,35,47,48] or solely provided a bolus before testing [17,24,31,37]. Loading phases ranged from 7 to 16 days; the ingestion time of boluses prior to exercise ranged from 60 to 120 minutes. One study utilizing a loading regime stated that on the day of testing, the final dose was ingested at the last meal before exercise [31]. Shanely et al. had participants consume the final dose of a loading regime during the time trial [35].

Five studies evaluated endurance performance using TTE as an outcome measure; the four remaining studies used TTC in various time-trial activities. Six studies used cycling for exercise testing, with three using running protocols. The source of funding was acknowledged in four out of the nine studies included, two of which had potential conflicts of interest [35,47].

### 3.3. Risk of bias within studies

Variable methodological quality resulted in the determination of a range of judgments in the risk of bias assessment, displayed in Table 2. Two studies were considered to have a low risk of bias, one a high risk of bias, and the remainders some concerns of bias. All studies claimed to randomize and conceal the allocation sequence, yet the majority provided no information regarding these methodological processes. Three studies described the method of allocation concealment [36,37,47]; two of which further detailed the randomization process for the allocation sequence [47]. Only two studies provided participant baseline information for allocation sequence groups, indicating no imbalances between groups with an equal number allocated to each [31,36].

All studies were double-blinded asides from one [35]; however, the introduction of performance bias was unlikely in this study with a resulting SMD of 0.04 favoring the control. Although double-blinding was reported, only four studies described the methodological procedures applied to blind participants, with one of these further stating how they blinded trial personnel. All studies allowed sufficient time to avoid carry-over effects and generally controlled well for confounding variables.

With regard to the inclusion of all randomized participants in the analyses, two studies did so [24,31]; three studies did not, and it was unclear in the remaining four studies so it was assumed all randomized participants were included in the analyses, [16,17,35,48]. All studies were considered to have a low risk of bias in the measurement of the outcome. Only one study specifically stated that outcome assessors were blinded to intervention allocation [36]. Shanely et al. provided no information regarding the blinding of outcome assessors. The remainder of the studies reported double-blinding so were assumed to have done so [35].

All studies were determined to have a low risk of bias regarding the selection of the reported result. Each study had a sole outcome measurement within the outcome domain, so authors could not selectively present one measurement over another based on the results. None of the studies used multiple analyses to produce a result for this outcome, and it is also unlikely that the authors selected results based on the identification of a carry-over effect. The risk of bias in individual studies is examined in more detail in the discussion section.

### 3.4. Results of individual studies

**Figure 2.**
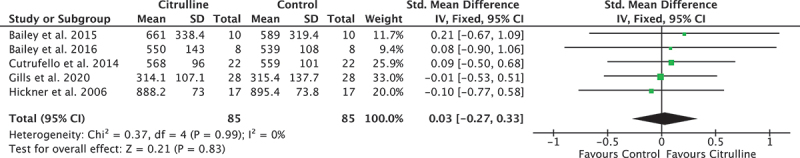
Forest plot for TTE data.

**Figure 3.**
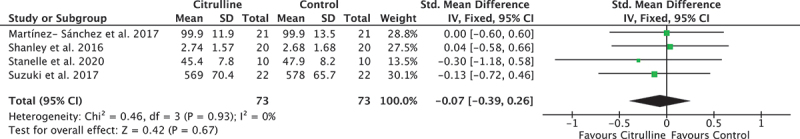
Forest plot for TTC data.

The mean and SD for the citrulline and control groups within each study are presented below. Alongside these are the SMD, corresponding 95% confidence intervals, and forest plots. The forest plot for studies measuring TTE is reported in Figure 2; the equivalent plot for those measuring TTC is reported in Figure 3. As previously stated, SE values were appropriately converted to SD to homogenize results and calculate the SMD. This conversion meant that the results of two studies reported to be significant [16,47], were no longer statistically significant. This is because the SE is a smaller value than the SD, so is commonly misused as a descriptive statistic to summarize the variability in their data making it appears more precise [40].

### 3.5. Synthesis of results

The 95% confidence intervals of the individual studies in each forest plot all overlap, suggesting that the degree of heterogeneity is small, and that meta-analysis was appropriate. Formal assessment using the chi-squared test revealed no significant heterogeneity in either comparison, for TTE (I^2^ = 0%, χ^2^ = 0.37, df = 4, *P* = 0.99) or TTC (I^2^ = 0%, χ^2^ = 0.46, df = 3, *P* = 0.93), validating the use of a fixed-effects analysis. The I^2^ statistic in both cases demonstrates a low risk of heterogeneity.

Neither plot shows a significant advantage to either the citrulline or control group. In Figure 2, three-point estimates are to the right of the vertical axis, one is to the left, and one crosses it. All 95% confidence intervals cross the line of no effect, indicating no study significantly favors one group over the other. The center of the diamond representing the point estimate of the pooled effect slightly favors the citrulline group. However, the width of the diamond representing the 95% confidence interval crosses the line of no effect. The pooled result for TTE data reinforces this (SMD = 0.03; *p* = 0.83), with the 95% confidence interval suggesting no statistically significant advantage to either group as it includes 0 (95% confidence interval = −0.27 to 0.33).

In Figure 3, two-point estimates are to the left of the vertical axis, and two cross it. Similarly, all 95% confidence intervals from individual studies cross the line of no effect. Although the point estimate of the pooled effect favors the citrulline group, the pooled 95% confidence interval also crosses the line of no effect. The pooled result for TTC data strengthens these findings (SMD = −0.07; *p* = 0.67), with a 95% confidence interval containing 0 showing that there is no statistically significant effect (95% confidence interval = −0.39 to 0.26). Overall, findings from the meta-analysis identified no significant benefit of citrulline when compared to control for the measures of endurance performance TTE and TTC.

### 3.6. Risk of bias across studies

As previously mentioned, no formal assessment for publication bias across studies was undertaken. However, no missing studies were found through inspection of trial registries, suggesting the risk was low. Studies reported all outcomes described within the domain of interest, making the introduction of bias through selective reporting unlikely.

### 3.7. Additional analyses

**Figure 4.**
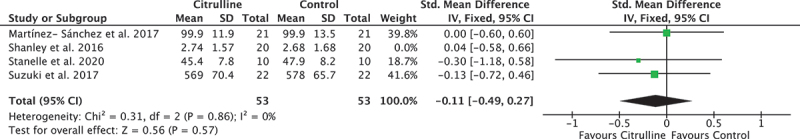
Post hoc sensitivity analysis of TTC outcome data.

The results of post hoc sensitivity analysis for the TTC outcome measure are displayed in Figure 4. This analysis excluded the study conducted by Shanely et al., judged to have a high risk of bias, to test the robustness of the originally conducted meta-analysis. Although the results of this analysis showed an increase in pooled effect size favoring citrulline (SMD = −0.11), this was still not statistically significant (*p* = 0.57; 95% confidence interval = −0.49 to 0.27).

## 4. Discussion

### 4.1. Summary of evidence

The null hypothesis was retained, meaning there was no significant difference in endurance performance after acute ingestion of citrulline compared with a control. A meta-analysis of the pooled data for TTE and TTC outcome measures found no significant difference in times between citrulline and control groups. Consequently, contrary to hypotheses, citrulline supplementation does not significantly improve the endurance performance measures TTE or TTC.

Our findings corroborate those of Gonzalez et al. [49]. These authors further specified that a single bolus dose of l-citrulline was not found to improve either TTE or time-trial exercise performance. However, it was suggested that a 7-day loading dosage regime may increase the likelihood of yielding positive outcome measures. They referenced two studies included in this review that used such a regime, although their results trended toward significantly favoring citrulline over a control [16,47], they were still not statistically significant. Other studies using a loading regime did not find a statistically significant difference either, further disagreeing with this hypothesis [35,36,48]. Fernández-Hernández et al. concluded following their systematic review that the ingestion of l-citrulline as an ergogenic aid may positively affect endurance performance but a lack of consistency across the results of their included studies prevented confirmation [33]. Only three of their included studies were incorporated into this review [16,31,47] due to differences in inclusion criteria. Therefore, regardless of the similarity in findings, comparisons drawn between these reviews should be measured.

### 4.2. Comparison with existing theories

#### 4.2.1. Nitric oxide production and vasodilation

Although theoretically robust, inconsistencies within the literature cast doubt on increase in NO production as the primary ergogenic mechanism. Five studies reported outcome data within this domain, with some finding that citrulline significantly increased plasma nitrate and nitrite concentrations (NO_x_) [16,35,48], and others reporting no significant change [31,47]. Bailey et al. proposed that when l-arginine increases NO synthesis a resultant ergogenic effect on exercise economy and performance is observed, yet when it does not increase NO synthesis no effect is observed [16]. The results of this review disagree, as regardless of changes in NO synthesis all other studies reported no significant effect on endurance performance. Suzuki et al. reported an increase in plasma arginine equal to that observed by Bailey et al.; the former found no significant increase in NO_x_ [47] but the later did [16]. This was attributed to poor sensitivity in the detection of NO biomarkers [47]. Previously, variations in citrulline dosage size, dosage regime, and participant training level have also been proposed to explain inconsistencies in eliciting this effect [32,37,50]. Well-trained individuals were presumed less likely to gain advantages due to greater resting circulatory NO levels than their sedentary counterparts [51].

Currently, there is limited evidence to suggest an association between increased plasma arginine levels and flow-mediated vasodilation in healthy individuals. Cutrufello et al. reported no change to resting brachial artery flow-mediated vasodilation 60–120 minutes after the ingestion of 6 grams of l-citrulline [24]. Future studies should measure bioactivity markers associated with NO-mediated vasodilation, such as urinary nitrate and cGMP, allowing for a more thorough examination of citrulline’s pharmacodynamic interactions [52,53].

Although no effect was found in this review, the ergogenic effects of citrulline supplementation have been demonstrated for other outcome domains in well-controlled studies with a low risk of bias [27]. This suggests that enhanced NO synthesis benefits anaerobic outcomes, not aerobic outcomes or that the beneficial effects of citrulline may occur through an alternative mechanism. In the former’s case, the quantity of muscle mass employed during exercise may offer a reason for the discrepancy in effect seen between these outcome domains.

Studies reporting an ergogenic effect tended to use anaerobic resistance exercise tasks isolating smaller groups of muscles [25,54,55]. Participants in this review were subject to endurance activities, involving gross utilization of large muscle groups. Vasodilation within skeletal muscle differs depending on the amount of muscle mass utilized during exercise in order to maintain sufficient mean arterial pressure (MAP) [56]. In small muscle mass exercises, overperfusion is more easily attainable without compromising MAP [56]. Prioritization of blood flow to more metabolically active areas of muscle is observed in whole-body exercise to protect MAP. The degree to which citrulline can affect blood flow may be more limited in large muscle mass exercises, such as running and cycling.

NO has also been reported to exert effects on exercise efficiency, mitochondrial respiration, calcium control in the sarcoplasmic reticulum, glucose uptake, and muscular fatigue [57]. However, the results of this review do not support these processes as primary mechanisms of action as they would all stand to benefit endurance exercise. Citrulline malate was found to accelerate the rate of phosphocreatine recovery after a finger flexion exercise [21]. This energy system is heavily utilized in high-intensity resistance exercise but not in aerobic endurance activities [58], perhaps offering an alternative mechanism to explain the discrepancy in findings between exercise types.

#### 4.2.2. Buffering of ammonia

Citrulline’s role in the urea cycle is another popular theory proposed to contribute to its ergogenic effects on exercise performance, supposedly prolonging the accumulation of lactate and impedance to muscular contraction [21]. Some findings in the literature discredit this as a chief mechanism of action. For example, numerous studies have reported ergogenic effects of citrulline supplementation on exercise in the absence of reduction to blood lactate levels [25,55,59]. Martínez-Sánchez et al. found that ingestion of watermelon juice enriched in l-citrulline 2 hours prior to a half marathon race reduced blood lactate levels but had no significant effect on performance time [17]. However, unless exercise intensity and duration are fixed, comparison of post-exercise blood lactate levels for citrulline supplementation and control may be misleading. This is because potential performance benefits from citrulline, such as greater exercise intensity or duration, may generate more lactate appearing to nullify the lactate clearing effect. In other words, the net effect on lactate levels may appear to be zero, even if the described mechanisms transpire and cause a performance benefit.

High-intensity strength and power exercises require a proportionately higher contribution from anaerobic glycolysis compared to endurance exercise [60]. Subsequently, rates of lactate accumulation observed may be higher and individuals may gain a larger benefit from ammonia buffering mechanisms, perhaps explaining the differences in performance effect in the literature. However, a systematic review by Rhim et al. in 2020 looked at 13 RCTs and concluded that citrulline supplementation significantly reduced post-exercise rating of perceived exertion and muscle soreness without affecting blood lactate levels [61].

#### 4.2.3. VO_2_ kinetics

In 2015, Bailey et al. reported that citrulline supplementation improved oxygen delivery to the muscle microvasculature, enabling a greater VO_2_ over the initial stages of a severe-intensity cycling exercise [16]. This suggests that citrulline increased the proportional energy contribution from oxidative metabolism [16]. Suzuki et al. found a correlation between plasma NO levels and power output/VO_2_ ratio after supplementation with citrulline; reporting greater power output for the same VO_2_ may have been related to improved plasma NO availability [47]. Bendahan et al. also found that citrulline malate supplementation increased muscle oxidative ATP production [21]. Nevertheless, in a later study Bailey et al. found no change in VO_2_ kinetics, which they suggested may have been due to the smaller dose of l-citrulline used. L-citrulline has not yet been reported to significantly affect VO_2_ max [24,31].

This evidence indicates citrulline potentially improves VO_2_ kinetics and exercise efficiency; further research is required to thoroughly investigate these mechanisms. Both studies eliciting these responses used a higher continuous dose of citrulline over at least seven days prior to exercise [16,47]. Studies focusing on such a dosage regime should be carried out in the future.

### 4.3. Strengths and limitations of the included studies

All participants were young healthy adults that volunteered to participate; however, the specific processes regarding participant recruitment are not detailed by any of the authors. Each study specifies inclusion criteria to ensure recruited participants represent the target population, reducing the risk of selection bias. However, due to the single centered nature and small sample sizes of the included studies, participants were likely selected from a small population in one location, reducing the generalizability of the results to the target population.

All included studies had a cross-over design, and the allocation sequences regarding the intervention and control were randomized in each trial, reducing the risk of allocation bias. Only two studies reported the method of randomization [36,47]; however, all but one study clearly concealed the allocation sequence generation. Therefore, it was reasonable to assume that the sequence was random for all studies but that performed by Shanely et al.

Only two studies provided baseline information to allow comparison between the two groups with opposing allocation sequences [31,36]. In both studies, these groups had an allocation ratio of 1:1, meaning period effects would cancel each other out [62]. Seven studies provided no information, preventing the assessment of any imbalances between the groups and therefore any influence from period effects. None of these studies provided information regarding the inclusion of period effects in their analysis either, leaving some concerns of allocation bias.

Shanely et al. introduced a risk of performance bias by not blinding participants or trial personnel to intervention allocation. However, measurement of the outcome was unlikely to be influenced by knowledge of the intervention received, so the risk of detection bias is low. All other studies reported double-blinding, reducing the risk of performance bias; however, only four described the methods used to ensure this. Only one study reported an attempt to assess blinding efficacy, asking participants to predict the allocation sequence [37]. However, the author did not report the results of this assessment. Only one study specifically reported blinding of outcome assessors, reducing the risk of detection bias. This was assumed to be the case for the remainder of the studies due to their double-blinded nature.

Two studies reported outcome data for all participants randomized [24,31], reducing the risk of attrition bias. One study excluded four individuals for not completing all of the exercise trials [37], the reasons for dropout were not reported. This was deemed to leave an insufficient amount of outcome data available for a continuous outcome with a small sample size, potentially introducing attrition bias. Stanelle et al. excluded one participant for not complying with their dietary protocol [36] and Suzuki et al. excluded three participants for having a cold on the day of testing [47]. Both of these studies were deemed to have enough outcome data to be confident of the findings. A general rule was followed that 90% of the randomized participants should be included in the analysis to be confident in the results [38]. Those studies that did not provide enough detail were assumed to have included all participants in their analysis [16,17,35,48]; however, the risk of attrition bias should still be considered.

The outcome measures, TTE and TTC, can only be measured one way with one measurement, reducing the risk of reporting bias. None of the studies used multiple methods of analysis, reducing the risk of bias that could be introduced through the selection of one or a subset of analyses. Studies conducting a paired analysis did not inappropriately present an unpaired analysis alongside to try and show an effect. No studies reported the outcomes of any statistical tests for carry-over; therefore, it is unlikely that first- or second-period data were reported alone. If this was the case, it would introduce bias if the two group’s baseline characteristics differed and remove the benefits of the cross-over design. No studies carried out post hoc analyses for the outcome domain of interest.

Potential confounders in these trials would include fatigue from recent exercise, differences in nutrient content of diet between exercise testing, as well as influence from alcohol, caffeine, and other supplementation on the results. The majority of studies controlled these factors well, asking participants to replicate their diet over the days preceding the exercise trials, as well as to abstain from alcohol, exercise, and other supplementation. Two studies asked participants to maintain their current exercise and dietary patterns, this may have introduced confounding bias [24,47]. Due to the strong cross-over designs and strict inclusion criteria individual characteristics were unlikely to affect the comparison between citrulline and the control. Familiarization testing was a feature in some studies to avoid any potential learning effect impacting performance and therefore the results. This was not practical for studies using an exercise protocol consisting of longer distances [17,35,36,47].

Only four provided details of their funding sources [17,35,36,47], two of these studies declared no conflicts of interest, reducing their risk of funding bias [17,36]. Suzuki et al. declared that two authors were employees of the company sponsoring their study [47], a conflict of interest potentiating funding bias. Similarly, Shanely et al. declared that one of their authors was a science advisor to the board funding the study. Although the funders may have had a vested interest in demonstrating a positive outcome, it was unlikely in this case as the study’s results slightly favored the control.

### 4.4. Generalizability of findings

The participants covered in this review are young healthy adults, the mean age reported in each study reflects the age of the population most likely to use this supplement [63]. Three of the included studies included populations of trained athletes [35,36,47], the remaining studies included participants that were only recreationally active. Elite athletes are most likely to utilize supplementation [63], in order to improve performance times by small margins. Therefore, most participants included in this review are not representative of the local population most likely to use citrulline.

None of the included studies used female-only samples; rendering the outcomes of this review unapplicable for this population. Cutrufello et al. studied a mixed population and reported no effect due to sex in their study [24]. However, it has been suggested that physiological function and response to supplementation may differ according to sex, due to the structural and morphological differences between males and females [64,65]. Consequently, it would be useful to study the effects of acute citrulline supplementation on endurance performance in female samples to identify any potential difference in outcomes.

As previously stated, the participant recruitment process was not reported. Therefore, the populations from which participants were selected is unknown, making it harder to comment on the generalizability of the results. Many studies used treadmills or cycle ergometers to complete exercise tasks; this is likely to differ from the settings and conditions usually endured by competitive endurance athletes. Only three studies observed athletes during a simulated competition over longer distances [17,35,36]. The remaining studies observed exercise tasks that may not have been representative of the distances usually undertaken by endurance athletes, perhaps reducing the generalizability of the results of this review.

### 4.5. Implications of the review

These results are relevant to many key groups. Athletes and nutritionists will be more aware of the benefits of citrulline supplementation and have an increased ability to make evidence-based judgments regarding its use for endurance exercise. The supplement industry will be able to more accurately market evidence-based benefits of citrulline, preventing them from misleading consumers. These findings are also beneficial to scientific and academic communities, providing a summary of the current evidence, its limitations, and gaps that would profit from further research.

Due to the limited number of studies, further research is required to identify any potential effect citrulline supplementation may have on endurance performance. Future research should focus on higher continuous doses of citrulline ingested over at least seven days before exercise. Endurance exercise tasks used should reflect simulated competition over longer distances. TTC outcomes imitate competitive endurance events more closely than TTE outcomes, so should be the primarily considered outcome measure. The establishment of core outcome sets in this research area should also be a priority to maximize criterion validity.

Elite athlete populations should be considered, as these individuals are most likely to use citrulline supplementation. Female populations should also be more extensively studied to identify any influence sex may have on the results. Potential underlying mechanisms requiring further investigation include citrulline’s impact on VO_2_ kinetics and exercise efficiency as well as the phosphocreatine energy system. More thorough reporting of methodological procedures within studies will allow a more accurate assessment of the risk of bias and increase the confidence in the conclusions drawn.

## 5. Conclusions

With the addition of two studies published in 2020, it was felt there was sufficient evidence surrounding citrulline supplementation and endurance performance to perform a systematic review and meta-analysis to pool and summarize the results. Two meta-analyses were performed as it was deemed inappropriate to combine the results of the two observed outcome measures, TTE and TTC. The results of the meta-analyses indicate that acute citrulline supplementation does not significantly improve the endurance performance measures, TTE (pooled SMD = 0.03 [−0.27, 0.33]) and TTC (pooled SMD = −0.07 [−0.39, 0.26]), in comparison to control in young healthy adults. The small number of included studies prevented further subgroup analyses. In the context of other evidence, these results are not surprising. Trexler et al. only found a small effect bordering on insignificance of citrulline supplementation on high-intensity power and strength outcomes in healthy men and women [27].

## Data Availability

Data from this meta-analysis is available and can be attained upon request from the corresponding author.
